# Brain volumes and regional cortical thickness in young females with anorexia nervosa

**DOI:** 10.1186/s12888-016-1126-9

**Published:** 2016-11-16

**Authors:** Tone Seim Fuglset, Tor Endestad, Eva Hilland, Lasse Bang, Christian Krog Tamnes, Nils Inge Landrø, Øyvind Rø

**Affiliations:** 1 Regional Department for Eating Disorders, Division of Mental Health and Addiction, Oslo University Hospital, Ullevål, Oslo Norway; 2 Department of Psychology, University of Oslo, Oslo, Norway; 3 Institute of Clinical Medicine, Division of Mental Health and Addiction, University of Oslo, Oslo, Norway

**Keywords:** Anorexia nervosa, Neuroimaging, Brain volumes, Cortical thickness, MRI, Adolescence

## Abstract

**Background:**

Anorexia nervosa (AN) is a severe mental illness, with an unknown etiology. Magnetic resonance imaging studies show reduced brain volumes and cortical thickness in patients compared to healthy controls. However, findings are inconsistent, especially concerning the anatomical location and extent of the differences. The purpose of this study was to estimate and compare brain volumes and regional cortical thickness in young females with AN and healthy controls.

**Methods:**

Magnetic resonance imaging data was acquired from young females with anorexia nervosa (*n* = 23) and healthy controls (*n* = 28). Two different scanner sites were used. BMI varied from 13.5 to 20.7 within the patient group, and 11 patients had a BMI > 17.5. FreeSurfer was used to estimate brain volumes and regional cortical thickness.

**Results:**

There were no differences between groups in total cerebral cortex volume, white matter volume, or lateral ventricle volume. There were also no volume differences in subcortical grey matter structures. However the results showed reduced cortical thickness bilaterally in the superior parietal gyrus, and in the right inferior parietal and superior frontal gyri.

**Conclusions:**

The functional significance of the findings is undetermined as the majority of the included patients was already partially weight-restored. We discuss whether these regions could be related to predisposing factors of the illness, or whether they are regions that are more vulnerable to starvation, malnutrition or associated processes in AN.

**Electronic supplementary material:**

The online version of this article (doi:10.1186/s12888-016-1126-9) contains supplementary material, which is available to authorized users.

## Background

Anorexia nervosa (AN) is a severe psychiatric illness characterized by extreme underweight, an intense fear of gaining weight, and a disturbance in the way one’s body weight or shape is experienced [1]. An increasing number of neuroimaging studies have investigated brain structure in patients with AN, but the majority of these studies has included adult samples. As the onset of AN usually is during adolescence [1], adults with AN will on average have a longer duration of illness than younger individuals. Due to starvation and malnutrition, it is likely that having an eating disorder over a long time period will affect the brain. To identify regions susceptible to short term starvation and malnutrition effects, it is of particular interest to investigate young people with AN.

Previous studies have typically reported reduced brain volumes in individuals with AN compared to healthy controls. Results from a recent meta-analysis showed that patients with AN had a significant decrease in total grey matter volume (GMV), white matter volume (WMV) as well as significantly increased cerebrospinal fluid (CSF) volumes [2]. In addition to reduced gross brain volumes and increased CSF in individuals with AN, regional grey matter volume differences between patients and controls have also been demonstrated. Findings are inconsistent, as the reported anatomical locations and extent of these differences are highly variable. The aforementioned meta-analysis reported that the hypothalamus, left inferior parietal gyrus, right lentiform nucleus and right caudate were decreased in AN [2]. Another meta-analysis aimed to quantify GMV, WMV and CSF alterations with regard to the time course of the disease and weight recovery [3]. They reported that global brain volume loss was more pronounced in adolescent patients than in adults. The authors also showed that following short-term weight recovery (about 4 months), about half of the GMV and CSF normalized, and the amount of WMV seemed to increase more rapidly. Regional analyses showed that some regions seemed to be more vulnerable to change, such as the hippocampus, cingulate gyrus and the midbrain.

To our knowledge, only three studies have investigated cortical thickness in patients with AN [4–6]. King et al. [4] found a significant widespread thinning of the cortex in adolescents and young adults with AN compared to healthy controls. They also investigated a group of individuals that had recovered from AN, and cortical thickness was not different between this group and the control group, suggesting that structural brain anomalies may be a consequence of malnutrition and is unlikely reflect premorbid traits. This was further supported in a later study [6], where the authors showed that cortical thickness in the ill AN group normalized following partial weight-restoration, illustrating the rapid reversal of cortical thinning. Lavagnino et al. [5] found reduced cortical thickness in women with AN, however their findings did not reach significance when correcting for multiple comparisons. They did however find a positive relationship between cortical thickness and body mass index (BMI) in these patients with less than two years of illness duration.

Findings of structural brain differences between patients with AN and healthy controls are inconsistent, especially concerning the anatomical location and extent of the differences. In addition, there is a paucity of studies of cortical thickness in AN. The purpose of this study was therefore to estimate and compare brain volumes and regional cortical thickness in young females with AN and healthy controls. We expected to find reduced brain volumes and cortical thickness in patients relative to controls.

## Methods

### Participants

Twenty-three females were recruited from an inpatient unit at the Regional Department for Eating Disorders in Oslo, Norway. This unit offers treatment at a tertiary level for patients suffering from an eating disorder, and all patients that participated in the current study received treatment for AN. The age range of the patients was 13–22 years (mean 17.4; SD, 2.2). AN diagnosis was determined at admission by a clinical evaluation performed by trained psychologists or psychiatrists, and according to the ICD-10 criteria. Weight gain is an important part of the treatment at the unit, and the patients follow a strict meal plan to avoid acute effects of malnutrition and dehydration, as well as to be more responsive to psychotherapeutic treatment. As the patients were in inpatient treatment, they varied in nutritional and weight status, and some were partially weight-restored. BMI varied from 13.5 to 20.7 within the patient group, and 11 patients had a BMI > 17.5. Twenty-eight healthy female control subjects, recruited from local schools, also participated in this study, with an age range from 15–23 years (mean = 17.6; SD = 2.2). For clinical measures, see Table 1.

**Table 1:** Results from independent sample t-tests of clinical measures

Characteristic	Patients (*n* = 23)	Controls (*n* = 28)		
	Mean (SD)	Mean (SD)	*P*-value	Cohen’s D
Age (years)	17.4 (2.2)	17.6 (2.2)	.674	0.1
Body mass index (BMI)	17.4 (2.0)	21.8 (2.8)	<0.001	1.8
BMI percentile	18.9 (13.1)	42.7 (18.2)	<0.001	1.5
Eating disorder symptoms (EDE-Q, Global score)	3.8 (1.2)	1.2 (0.9)	<0.001	2.5
Depression (BDI)	31.1 (10.2)	7.9 (7.6)	<0.001	2.6
State anxiety (STAI)	55.3 (12.9)	32.1 (10.3)	<0.001	2.0
Trait anxiety (STAI)	60.4 (7.9)	37.1 (11.3)	<0.001	2.4

Results from independent sample t-tests testing for group differences in clinical measures between patients with anorexia nervosa and healthy controls

*EDE-Q* Eating Disorder Examination Questionnaire, *BDI* Beck’s Depression Inventory, *STAI* State-Trait Anxiety Inventory

### Clinical measures

The Eating Disorders Examination Questionnaire (EDE-Q) provides a comprehensive assessment of specific eating disorder behavior [7]. It is a 28-item self-report questionnaire and measuring eating disorder psychopathology the past 28 days. It produces an overall global score, and four sub-scores. Only global scores are presented in the current paper. The items are rated on a 7 –point scale, and higher scores indicate increased eating disorder psychopathology.

State/Trait Anxiety Inventory (STAI) is a commonly used measure of trait and state anxiety [8]. STAI is a self-report measure, and has 20 items for assessing trait anxiety and 20 items for assessing state anxiety. All items are rated on a 4 - point scale, where higher scores indicate increased anxiety.

Beck’s Depression Inventory (BDI) is a 21-item self-report questionnaire and is used to measure severity of depression [9]. The items are related to various symptoms of depression, such as hopelessness and irritability, cognitions of guilt, or feelings of being punished. Higher scores indicate increased depressive symptoms.

### Image acquisition

Scans were acquired on two different scanners with different sequences. The first scanner was a 1.5 T General Electric Healthcare Signa scanner, with an 8-channel head coil and a coronal 3D FSPGR sequence with the following parameters: repetition time (TR) = 21000 ms, echo time (TE) = 6.00 ms, flip angle (FA) = 35°, field of view (FOV) = 280 mm, and1.09 × 1.09 × 1.7 mm voxels. Images from nineteen patients and twelve controls were acquired at this scanner.

The second scanner was a 1.5 T Siemens Avanto, with a 12-channel head coil and a sagittal 3D MPR sequence with the following parameters: TR = 1940 ms, TE = 3.09 ms, FA = 15°, FOV = 256 mm, and 1.0 × 1.0 × 1.0 mm voxels. Images from four patients and sixteen controls were acquired at this scanner. Descriptive statistics for patients and controls at scanner 1 and scanner 2 separately is presented in Additional file 1: Table S1.

### MRI analysis

The raw MRI data were converted to the NIFTI format using NordicICE software (NordicNeuroLab, Bergen, Norway, http://www.nordicneurolab.com). Volumetric segmentation and cortical surface reconstruction was performed with the FreeSurfer image analysis suite (version 5.1.; Athinoula A. Martinos Center for Biomedical Imaging, Boston, MA) (http://freesurfer.net/). The technical details are described in prior publications [10, 11]. The processing stream includes motion correction, removal of non-brain tissue, automated Talairach transformation, intensity correction, volumetric segmentation, cortical surface reconstruction, and parcellation*.*

All MRI volumes were quality checked, and manual edits (removal of non-brain tissue included within the cortical boundary) were performed by a trained operator. Cortical surface maps were resampled, and mapped to an average surface, smoothed using a Gaussian kernel with a full-width at half-maximum of 15 mm, and submitted to statistical analyses. One patient and two control participants were excluded from the data analyses due to data artefacts (i.e., excessive movement during scanning). A total of twenty-eight controls and twenty-three patients were included in the analyses.

### Statistical analyses

Independent sample *t*-tests in Statistical Package for the Social Sciences (SPSS) were used to compare demographic and clinical measures between the patient group and the control group.

To investigate between-group differences in cerebral cortex volume, cerebral white matter volume, lateral ventricles volume as well as subcortical GMV, a series of linear regression analyses were carried out. Group was inserted as an independent variable in all models, and scanner was included as a covariate.

Whole-brain surface-based cortical analyses were performed on a vertex-wise (point-by-point) level using general linear models (GLMs), as implemented in FreeSurfer. Main effects of group were tested by contrasting the AN patients and controls while controlling for scanner. The data were tested against an empirical null distribution of maximum cluster size across 10,000 iterations using Z Monte Carlo simulation as implemented in Freesurfer [12, 13] synthesized with a cluster-forming threshold of *p* < .05 (two-sided), yielding clusters fully corrected for multiple comparisons across the surfaces. Clusterwise corrected p (CPW) < .05 was regarded as significant. Significant regions highlighted in the statistical difference maps were neuroanatomically identified based on the Desikan-Killiany cortical atlas [14]. Mean vertex-wise cortical thickness values were extracted from each clusters showing a significant difference between the patients with AN and the controls. Follow-up analyses were performed to test whether the observed differences in cortical thickness between patients and controls were affected by scanner by performing linear regression analyses in SPSS comparing controls from scanner 1 (*n* = 12) and controls from scanner 2 (*n* = 16), with age included in the analysis as covariate.

Finally, to test whether observed differences in cortical thickness at the group level were related to body mass index (BMI) percentiles within the group of patients with AN, we performed partial correlations in SPSS between mean cortical thickness in each cluster and BMI, controlling for scanner and age.

## Results

### Sample characteristics

The patient group and the healthy control group did not differ significantly with respect to age. As expected, BMI and BMI percentiles differed significantly between the AN and the control group. Also, patients had significantly higher scores on eating disorder symptoms, depression and anxiety, than the control group (see Table 1).

### Gross brain volumes

Average total cerebral cortex volume, cerebral white matter volume and lateral ventricles volume were estimated for each group and presented in Table 2. Linear regression analyses, controlling for scanner, revealed no significant differences between the patient group and the control group.

**Table 2:** Results from linear regression analyses showing associations between group and cerebral cortex volume, cerebral white matter volume and lateral ventricles volume

	Patients (*n* = 23)	Controls (*n* = 28)	Unstandardized coefficients		Standardized coefficient	
	Mean (SD)	Mean (SD)	B	St error	Beta	Sig
Cerebral cortex volume	484307.1 (47284.0)	505289.5 (62943.2)	5115.9	16621.4	.045	.760
Cerebral white matter volume	467243.7 (51329.4)	458881.8 (53414.5)	−19220.4	15858.6	−.185	.231
Lateral ventricles volume	12273.1 (6654.2)	11424.3 (5967.4)	−1561.9	1938.0	−.126	.424

Results from linear regression analyses testing for group differences in cerebral cortex volume, cerebral white matter volume and lateral ventricles volume between patients with anorexia nervosa and healthy controls. Scanner was included in the analyses as covariate. Volumes are in mm^3^. Total cerebral cortex volume, volume inside the pial surface, minus tissue inside the ribbon that is not a part of the cortex; cerebral white matter volume, volume inside the white surfaces; lateral ventricles volume, combined left and right lateral and inferior lateral ventricles

### Gross brain volumes and BMI

Partial correlations were run to determine the relationship between gross brain volumes and BMI within the patient group while controlling for scanner and age. There were no significant correlations between BMI percentiles and cortex volume (*r* = −.339, *p* = .143), cerebral white matter (*r* = −.132, *p* = .580) and lateral ventricles (*r* = −.423, *p* = .063).

### Subcortical grey matter volumes

Average subcortical GMVs were estimated for each group and presented in Table 3. Linear regression analysis, controlling for scanner, revealed no significant group differences in the subcortical GMVs, which included thalamus, caudate, putamen, pallidum, hippocampus and amygdala.

**Table 3:** Results from linear regression analyses showing associations between group and subcortical grey matter volumes

		Patients (*N* = 23)	Controls (*N* = 28)	Unstandarized coefficient			
	Region label	Mean (SD)	Mean (SD)	B	St Error	Stand.beta	Sig
Left hemisphere	Thalamus	7063.9 (785.7)	7568.8 (916.5)	85.5	221.9	.048	.702
	Caudate	3702.3 (512.5)	3799.5 (494.2)	−23.0	150.3	−023	.879
	Putamen	5667.3 (539.6)	5716.7 (730.3)	40.5	202.6	.032	.842
	Pallidum	1728.0 (216.3)	1802.6 (271.7)	45.1	76.5	.091	.559
	Hippocampus	4216.2 (506.6)	4188.9 (276.1)	−58.1	122.8	−.074	.638
	Amygdala	1492.1 (207.8)	1458.0 (185.2)	−59.9	60.3	−.147	.350
Right hemisphere	Thalamus	7152.5 (705.3)	7710.3 (858.3)	183.5	207.6	.111	.381
	Caudate	3696.8 (512.4)	3795.0 (491.0)	−54.0	146.0	−.054	.731
	Putamen	5417.9 (543.8)	5414.3 (671.6)	−83.0	189.9	−.068	.664
	Pallidum	1525.7 (215.4)	1661.7 (260.2)	68.8	71.1	.139	.338
	Hippocampus	4259.5 (283.6)	4229.8 (336.7)	−105.6	93.8	−.170	.266
	Amygdala	1558.4 (232.7)	1554.1 (217.4)	−17.0	69.6	−.038	.809

Results from linear regression analyses testing for group differences of subcortical grey matter volumes between patients with anorexia nervosa and healthy controls. Scanner was included in the analyses as covariate. Volumes are in mm^3^

### Subcortical grey matter volumes and BMI

Using partial correlations, there were no significant correlations between subcortical grey matter volumes and BMI percentiles (*r* = .021 – *r* = .173, *p* = .355 – *p* = .929) except between left putamen and BMI percentiles (*r* = .460, *p* = .041).

### Cortical thickness

**Fig. 1:**
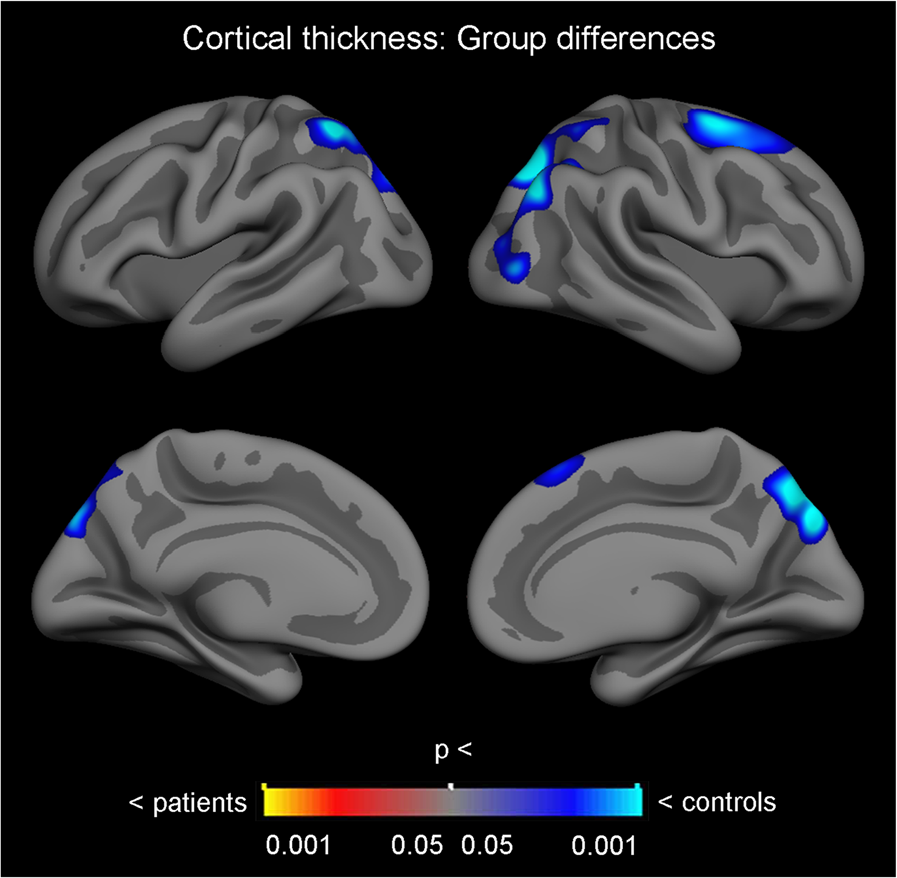
Results from GLM whole surface vertex-wise between-group analysis of cortical thickness, showing reduced cortical thickness in the patient group compared to the control group. Clusterwise CWP < .05

Group differences in cortical thickness between individuals with AN and healthy controls were tested with GLMs, while controlling for scanner (CWP < 0.05). The results showed four significant clusters, with maximum effects located in the left superior parietal gyrus, the right superior parietal gyrus, the right inferior parietal gyrus and the right superior frontal gyrus. The results are presented in Table 4, see also Fig. 1. All clusters showed negative effects, indicating group-level thinner cortex in patients relative to the controls. No effects were seen in the opposite direction. For further exploring the effect of scanner, linear regression analyses were performed, comparing cortical thickness for control participants from scanner 1 and control participants from scanner 2 within each of the four significant clusters. Age was included as a covariate. Results showed no significant differences; see Additional file 2: Table S2. To further investigate the anatomical specificity to the results, the main analysis testing for group differences in cortical thickness between individuals with AN and healthy controls (while controlling for scanner) was repeated with a more rigorous threshold (CWP < 0.01). Two of the four clusters remained significant (see Additional files 1, 2, 3 and 4, Fig. 1 and Table 3).

**Table 4:** Results from GLM whole surface vertex-wise between-group analysis of cortical thickness

	Annotation max vertex	MNI coordinates max vertex (x, y, z)			Size (mm^2^)	CWP
Left hemisphere	Superior parietal gyrus	−25.1	58.6	58.1	1766.95	0.016
Right hemisphere	Superior parietal gyrus	18.8	−71.1	44.1	2402.59	0.002
	Inferior parietal gyrus	41.1	−71.6	35.5	2218.18	0.004
	Superior frontal gyrus	23.1	0.5	62.2	1674.74	0.026

Results from GLMs testing for group differences in cortical thickness between patients with anorexia nervosa and controls, controlling for scanner, and with simulation-based clusterwise correction for multiple comparisons. All clusters showed negative associations, indicating reduced cortical thickness in the patient group compared to the control group. Clusterwise CWP < .05

### Cortical thickness and BMI

Partial correlations were run to determine the relationship between cortical thickness and BMI within the patient group whilst controlling for scanner and age. There were no significant correlations between BMI percentiles and mean thickness in the left superior parietal gyrus cluster (*r* = .018, *p* = .942), the right superior parietal gyrus cluster (*r* = −.078, *p* = .751), the right inferior parietal gyrus cluster (*r* = .171, *p* = .484), and the right superior frontal gyrus cluster (*r* = −102, *p* = .679).

## Discussion

This study aimed to investigate brain volumes and regional thickness in young females with AN. Our findings revealed regionally lower cortical thickness in patients with AN compared to the healthy control group. There were no significant differences in total cerebral cortex volume, cerebral white matter volume, lateral ventricles volume, or subcortical grey matter volumes between the groups.

Our results are in contrast to previous studies reporting significantly reduced grey and white matter volumes [15–17]. However, our findings support previous studies that have reported no differences in measures of total brain volume between patients with AN and controls, although this should be investigated further, as the sample sizes of this and several of the previous studies on this patient population have been relatively small. However, it is important to note that the null-findings in the present and previous studies may be related to low statistical power, and the fact that the majority of patients was already partially weight-restored. Some studies have found, in adolescents with AN, alterations in GMV and CSF, but not in WMV [18, 19]. Another study, which included adult women with AN, found no significant differences in total GMV, total WMV, or CSF [20]. Another study including adult women with AN, found that total brain volume was similar in patients and healthy controls [21].

Our investigation of subcortical GM volumes revealed no significant differences between individuals with AN and healthy controls. Findings are similar to previous studies, who also failed to show significant reductions of subcortical GM volumes [21–23]. However, previous studies have also reported subcortical volume reductions in adolescent patients compared to controls in the nucleus accumbens, amygdala, cerebellum, hippocampus, putamen and thalamus [4], while studies including adult patients with AN, have shown decreased GM volume of the amygdala and putamen [24], hypothalamus, caudate nucleus [15], hippocampus, [25], and the hippocampus-amygdala formation [26]. Discrepancies in findings related to global and subcortical brain volumes could be due to the heterogeneity in sample characteristics such as severity of emaciation or treatment duration.

We found regionally lower cortical thickness bilaterally in the superior parietal gyrus, in the right inferior parietal gyrus and in the right superior frontal gyrus in patients with AN compared to controls. Regional brain alterations seems to vary considerably across studies, however, reduced GMV in the inferior and superior parietal lobes has previously been reported in an adolescent sample with restrictive type AN [16]. Reduced volume in the left inferior parietal lobe in AN was also reported in the meta – analysis by Titova et al., however in the left hemisphere [2]. Our results are modest compared to the results from King and colleagues [4], who reported widespread cortical thinning, with significantly thinner cortex in 86% of the cortical surface in patients with AN (*n* = 40) compared to healthy controls (*n* = 34). In that study, only two regions showed no group differences: the bilateral temporal pole and the entorhinal cortex. The discrepancy between the results from the King et al. study and our own might be due to statistical power, as the former had a larger sample size. Indeed, in a more recent study [5], differences in cortical thickness between patients (*n* = 21) and controls (*n* = 18) did not survive correction for multiple comparisons, and the authors suggested that findings might have survived with a larger sample. Alternatively, it is possible that our results did not show more extensive cortical thinning due to the heterogeneity of BMI and treatment duration. It was recently shown that the reductions in cortical thickness of AN patients normalize rapidly following partial weight-restoration [6]. As there was some variability in the treatment duration and BMI of the patients included in our study, some degree of cortical normalization may already have occurred.

Our results showed no correlation between BMI and cortical thickness in the clusters found to have lower cortical thickness in patients than controls. These findings are consistent with those of King et al. [4] and Bär et al. [27], however in contrast to findings from Seitz et al. [3] and Lavagnino et al. [5]. Inconsistencies in these findings could be related to methodological variabilities, such as sample characteristics including age, duration of illness and nutritional status.

Whether the brain completely normalizes with recovery, is still under debate. Longitudinal studies and studies including individuals that have recovered from AN have been performed to investigate whether the brain normalize with recovery and weight restoration. Results from a recent meta-analysis [3] show that in acute AN, GMV was reduced by 5.6% and WMV by 3.8% compared to healthy controls. Short-term weight recovery (2.5 months after admission) led to restitution of about half of the GM loss, and almost full WM recovery. After 2–8 years of remission, GMV and WMV were approximately normalized. In the study by Bernardoni et al. [6] patients had normal cortical thickness after recovery, which supports the notion that differences in cortical thickness are related to weight. On a case level, it has been demonstrated that increased weight is associated with increased GMV, and likewise, reduced weight is associated with reduced GMV [28]. On the other hand, some studies have found persistent brain alterations in AN [18, 19].

There are some limitations in this study. First, since patients were scanned at different times during the inpatient treatment period, there was considerable heterogeneity in our sample of adolescent AN patients in terms of BMI. As weight gain is a major focus at the initiation of treatment of severely underweight patients, there is a possibility that this might have led to partial restoration of brain tissue. It would have been useful to include prior weight loss of the patients as well as degree of weight recovery in the analyses, however this information was not available in the current study. Second, two different scanners with different sequences were used, and it is generally recommended to, if possible, avoid mixing scans from different scanners and protocols. Critically, patient and control participants were not distributed proportionally across these two scanners, and this represents a caveat for the interpretation of the results. However, scanner was included as a covariate in all analyses, and follow-up analyses were performed to test the effects of these data acquisition differences. Supplementary data provides demographic and clinical measures for patients and controls for each scanner site, see Additional file 1: Table S1. Furthermore, Additional file 2: Table S2 presents the results of a linear regression analysis testing for group differences in cortical thickness between controls from scanner 1 and scanner 2. There were no significant differences between the groups. A third limitation is the variability of age within the groups. Unfortunately, due to insufficient statistical power, it was not possible to investigate the effect of age and the interaction effect of age and group. However, there were no significant differences in mean age between the groups. Finally, information regarding pharmacological treatment was not available and therefore not investigated in our analyses.

## Conclusions

To conclude, our study showed that young patients with AN have regional cortical thickness reductions in parietal and frontal areas. We did not find any reductions of global GM volume or subcortical GM volumes, however, this could be explained by the fact that some of the patients were partially weight restored. Future studies should aim to understand the large variability in brain structure alterations in AN across studies. It is possible that degree of weight restoration might explain a large part of the variance, and should be further explored. It would also be of interest to investigate whether the apparent reduced brain volumes and cortical thickness is linked to the symptoms and behavior seen in AN, or whether these brain alterations are merely global consequences of underweight and malnutrition. In addition, the mechanisms underlying the reduction of brain tissue volumes and cortical thickness in AN is not known. Bernardoni et al. [6] ruled out colloidal osmotic pressure of blood and dehydration as possible mechanisms underlying cortical thinning, however more studies investigating this field is warranted.

## Additional files

Additional file 1: Table S1. Descriptive statistics for patients and controls from scanner 1 and scanner 2. Descriptive statistics (age, BMI, EDE-Q, BDI, STAI state and STAI trait) for patients and controls from scanner 1 and scanner 2. (DOCX 14 kb)
Additional file 2: Table S2. Associations between scanner and cortical thickness in controls. Results from linear regression analyses testing for group differences in cortical thickness between controls from scanner 1 and controls from scanner 2. Age was included in the analyses as covariate. Average thickness in each cluster is in mm. (DOCX 14 kb)
Additional file 3: Figure S1. Results from GLM whole surface vertex-wise between-group analysis of cortical thickness, showing reduced cortical thickness in the patient group compared to the control group. Clusterwise CWP < .01. (TIF 3413 kb)
Additional file 4: Table S3. Results from GLM whole surface vertex-wise between-group analyses of cortical thickness. Results from GLMs testing for group differences in cortical thickness between patients with anorexia nervosa and controls, controlling for scanner, and with simulation-based clusterwise correction for multiple comparisons at *p* < .01. All clusters showed negative associations, indicating reduced cortical thickness in the patient group compared to the control group. (DOCX 14 kb)

## Abbreviations

AN: Anorexia nervosa
BDI: Beck’s Depression Inventory
BMI: Body mass index
CPW: Clusterwise corrected p
CSF: Cerebrospinal fluid
EDE-Q: Eating disorder examination questionnaire
FA: Flip angle
FOV: Field of view
GLMs: General linear models
GMV: Grey matter volume, MRI, magnetic resonance imaging
SPSS: Statistical package for social sciences
STAI: State/trait anxiety inventory
TE: Echo time
TR: Repetition time
WMV: White matter volume
