# Supplementary material for: Brain volumes and regional cortical thickness in young females with anorexia nervosa
Source: BMC Psychiatry. 2016 Nov 16;16:404. doi: 10.1186/s12888-016-1126-9 (PMC5112631; doi:10.1186/s12888-016-1126-9)
Supplement: Additional file 1: Table S1. — Descriptive statistics for patients and controls from scanner 1 and scanner 2. Descriptive statistics (age, BMI, EDE-Q, BDI, STAI state and STAI trait) for patients and controls from scanner 1 and scanner 2. (DOCX 14 kb) [file 12888_2016_1126_MOESM1_ESM.docx]

| **Table 1:** Descriptive statistics for patients and controls from scanner 1 and scanner 2 | | | | |
| --- | --- | --- | --- | --- |
|  | **Scanner 1** | | **Scanner 2** | |
|  | **Patients (n=19) Mean (SD)** | **Controls (n=12) Mean (SD)** | **Patients (n=4) Mean (SD)** | **Controls (n=16) Mean (SD)** |
| **Age** | 17.17 (2.16) | 15.90 (0.77) | 18.23 (2.22) | 18.9 (1.97) |
| **BMI** | 16.78 (4.49) | 22.61 (3.60) | 16.25 (1.62) | 21.31 (2.01) |
| **EDE-Q** | 3.93 (1.13) | 1.30 (0.84) | 3.19 (1.66) | 1.04 (0.94) |
| **BDI** | 33.93 (8.23) | 8.08 (5.26) | 20.25 (10.28) | 7.88 (9.22) |
| **STAI state** | 56.36 (13.85) | 34.45 (7.83) | 51.75 (9.25) | 30.38 (11.59) |
| **STAI trait** | 60.60 (8.32) | 40.82 (8.07) | 59.50 (7.14) | 34.50 (12.66) |
