# Supplementary material for: Brain volumes and regional cortical thickness in young females with anorexia nervosa
Source: BMC Psychiatry. 2016 Nov 16;16:404. doi: 10.1186/s12888-016-1126-9 (PMC5112631; doi:10.1186/s12888-016-1126-9)
Supplement: Additional file 2: Table S2. — Associations between scanner and cortical thickness in controls. Results from linear regression analyses testing for group differences in cortical thickness between controls from scanner 1 and controls from scanner 2. Age was included in the analyses as covariate. Average thickness in each cluster is in mm. (DOCX 14 kb) [file 12888_2016_1126_MOESM2_ESM.docx]

| **Table 2.** Associations between scanner and cortical thickness in controls | | | | | | |
| --- | --- | --- | --- | --- | --- | --- |
| **Annotation max vertex** | **Controls scanner 1 (n=12)** | **Controls scanner 2 (n=16)** | **Unstandarized coefficient** | |  |  |
|  | **Mean (SD)** | **Mean (SD)** | **B** | **St Error** | **Stand.beta** | **Sig** |
| Left superior parietal gyrus | 2.468 (.220) | 2.621 (.189) | .061 | .105 | .145 | .566 |
| Right superior parietal gyrus | 2.404 (.210) | 2.494 (.197) | -.015 | .104 | -.037 | .886 |
| Right inferior parietal gyrus | 2.818 (.206) | 2.766 (.190) | -.180 | .099 | -.466 | .080 |
| Right superior frontal gyrus | 3.093 (.291) | 3.050 (.167) | -.046 | .122 | -.103 | .709 |
| *Notes:* Results from linear regression analyses testing for group differences in cortical thickness between controls from scanner 1 and controls from scanner 2. Age was included in the analyses as covariate. Average thickness in each cluster is in mm. | | | | | | |
