# Supplementary figures and images for: Brain volumes and regional cortical thickness in young females with anorexia nervosa
Source: BMC Psychiatry. 2016 Nov 16;16:404. doi: 10.1186/s12888-016-1126-9 (PMC5112631; doi:10.1186/s12888-016-1126-9)

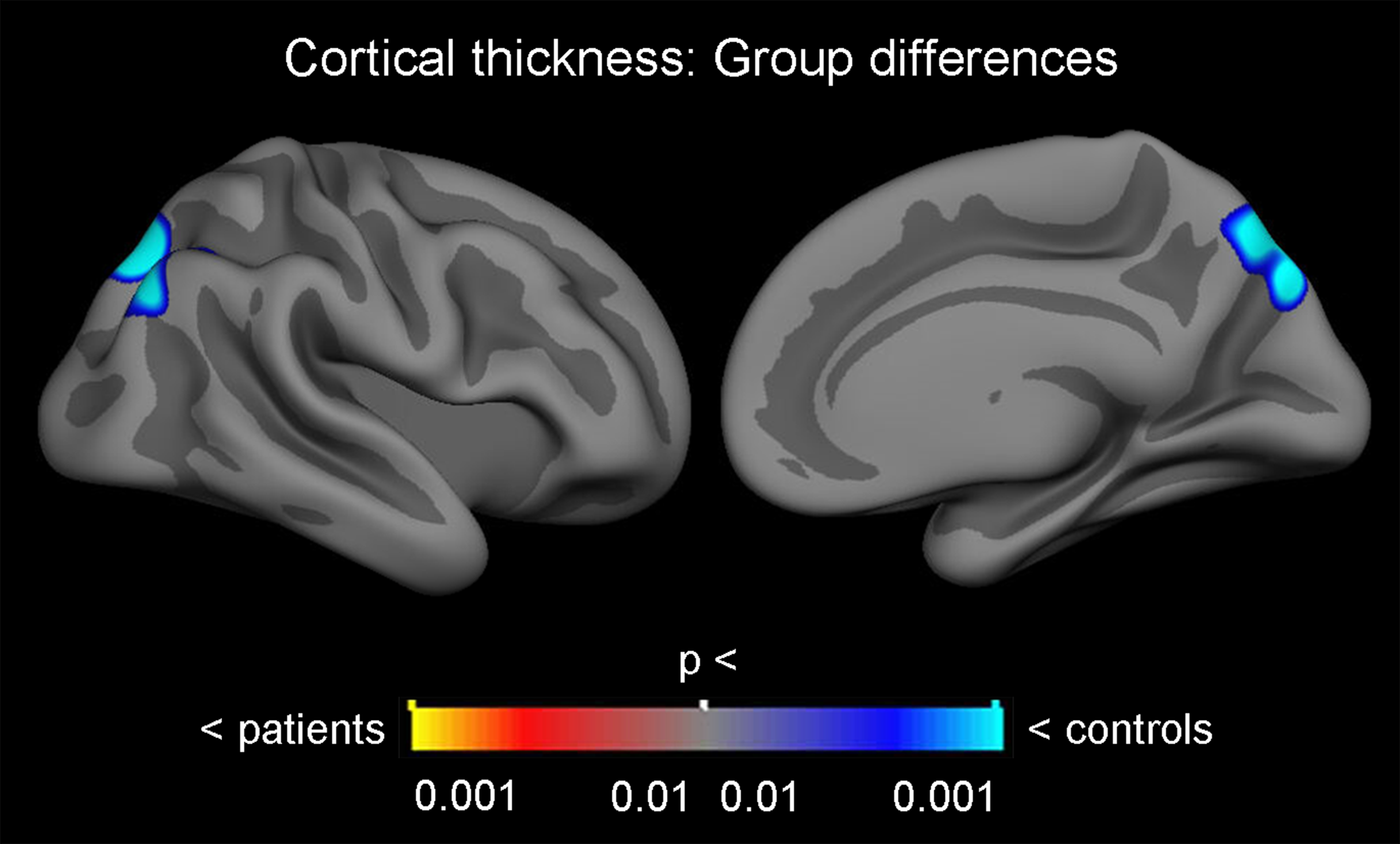

Supplement: Additional file 3: Figure S1. — Results from GLM whole surface vertex-wise between-group analysis of cortical thickness, showing reduced cortical thickness in the patient group compared to the control group. Clusterwise CWP < .01. (TIF 3413 kb) [file 12888_2016_1126_MOESM3_ESM.tif]
