# Supplementary material for: Brain volumes and regional cortical thickness in young females with anorexia nervosa
Source: BMC Psychiatry. 2016 Nov 16;16:404. doi: 10.1186/s12888-016-1126-9 (PMC5112631; doi:10.1186/s12888-016-1126-9)
Supplement: Additional file 4: Table S3. — Results from GLM whole surface vertex-wise between-group analyses of cortical thickness. Results from GLMs testing for group differences in cortical thickness between patients with anorexia nervosa and controls, controlling for scanner, and with simulation-based clusterwise correction for multiple comparisons at p < .01. All clusters showed negative associations, indicating reduced cortical thickness in the patient group compared to the control group. (DOCX 14 kb) [file 12888_2016_1126_MOESM4_ESM.docx]

| **Table 3.** Results from GLM whole surface vertex-wise between-group analysis of cortical thickness | | | | | | |
| --- | --- | --- | --- | --- | --- | --- |
|  | **Annotation max vertex** | **MNI coordinates max vertex (x, y, z)** | | | **Size (mm^2^)** | **CWP** |
| **Right hemisphere** | Superior parietal | 18.8 | -71.1 | 44.1 | 1432.97 | < 0.001 |
|  | Inferior parietal | 41.1 | -71.6 | 35.5 | 676.16 | 0.047 |
| *Notes*: Results from GLMs testing for group differences in cortical thickness between patients with anorexia nervosa and controls, controlling for scanner, and with simulation-based clusterwise correction for multiple comparisons at p < .01. All clusters showed negative associations, indicating reduced cortical thickness in the patient group compared to the control group. | | | | | | |
